# Rhythmic Changes in Gene Activation Power the Circadian Clock

**DOI:** 10.1371/journal.pbio.1001443

**Published:** 2012-11-27

**Authors:** Janelle Weaver

**Affiliations:** Freelance Science Writer, Glenwood Springs, Colorado, United States of America

**Figure:**
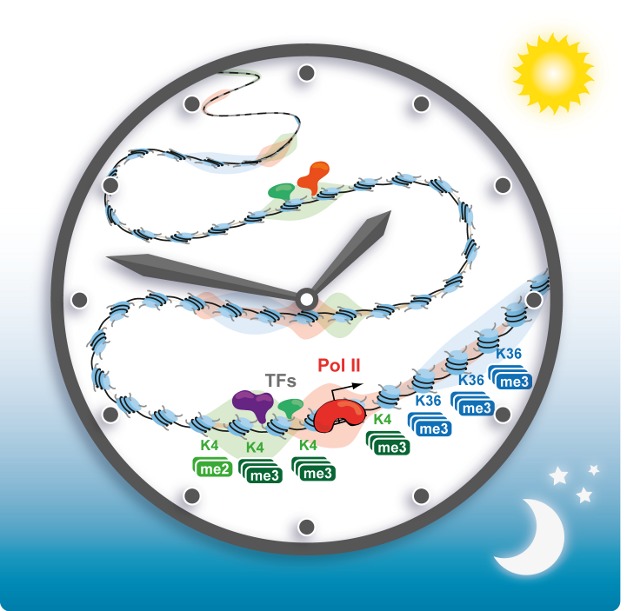
Daily rhythms in mRNA polymerase II transcription and histone modifications influence gene expression programs responsible for the temporal gating of liver physiology and metabolism in mammals. Image Credit: Laura Symul (EPFL).

Rhythms underlie the daily functions of mammals, from sleep-wake cycles to metabolic processes in the liver. The circadian clock has evolved in response to daily changes in temperature and light in the environment. At the root of circadian rhythms are daily fluctuations in gene expression, which occur in part through the process of transcription—the creation of RNA from sequences of DNA. Although past studies have uncovered how changes in transcription states relate to irreversible processes, for example when cells become more specialized, much less is known about how transcription fluctuates in synch with recurring cycles.

In this issue of *PLOS Biology*, new insights into the dynamic nature of transcription are provided in a study led by Nouria Hernandez of the University of Lausanne and Felix Naef of the Ecole Polytechnique Fédérale de Lausanne. The findings reveal the kinetics by which genes are activated in a rhythmic manner as well as the remarkable impact of daily cycles across the genome.

In the study, Hernandez and Naef used a combination of experimental and computational methods to study genome-wide changes in transcription in the mouse liver, and how these changes relate to messenger RNA (mRNA) levels. They discovered that transcription in the liver occurs predominantly in morning and evening waves. These waves were accompanied by the rhythmic recruitment of RNA polymerase II (Pol II)—an enzyme that catalyzes transcription—to DNA strands. In addition, there were rhythmic changes in the modifications of histones—proteins that act as a scaffold for DNA and help to regulate gene expression. These rhythmic changes in transcription were driven primarily by the recruitment of Pol II to DNA.

The researchers also identified three classes of genes. One class showed both rhythmic transcription and mRNA fluctuations, a second class showed rhythmic transcription but flat mRNA levels, and a third class showed constant transcription but rhythmic mRNA fluctuations. The latter finding—that the levels of some mRNAs oscillated even when transcription remained constant—suggests that transcription alone does not regulate all rhythmic changes in mRNA levels. Instead, other processes, such as daily fluctuations in the rate of mRNA degradation, influence the accumulation of mRNA. These results indicate that molecular events taking place after transcription play a greater role in regulating daily fluctuations in mRNA levels than previously thought.

Taken together, the study reveals that many functions in the liver, such as lipid and carbohydrate metabolism as well as detoxification, are under the control of rhythmic changes in transcription. The findings could lead to insights into how the daily cycle influences genomic responses to food intake, eventually paving the way to the development of novel treatment strategies for diabetes and other metabolic diseases.

**Le Martelot G, Canella D, Symul L, Migliavacca E, Gilardi F, et al. (2012) Genome-Wide RNA Polymerase II Profiles and RNA Accumulation Reveal Kinetics of Transcription and Associated Epigenetic Changes During Diurnal Cycles. doi:10.1371/journal.pbio.1001442**

